# Spontaneous angiogram-negative subarachnoid hemorrhage: a retrospective single center cohort study

**DOI:** 10.1007/s00701-021-05069-7

**Published:** 2021-12-01

**Authors:** Alexander Achrén, Rahul Raj, Jari Siironen, Aki Laakso, Johan Marjamaa

**Affiliations:** 1 Department of Neurosurgery, University of Helsinki and Helsinki University Hospital, Töölö Hospital, HUS, Topeliuksenkatu 5, PO. Box. 266, 00029 Helsinki, Finland; 2 Division of Clinical Neuroscience, Department of Neurosurgery, University of Oulu, Oulu, Finland

**Keywords:** Subarachnoid hemorrhage, Perimesencephalic subarachnoid hemorrhage, Intensive care unit, Spontaneous subarachnoid hemorrhage

## Abstract

**Background:**

Spontaneous angiogram-negative subarachnoid hemorrhage (SAH) is considered a benign illness with little of the aneurysmal SAH-related complications. We describe the clinical course, SAH-related complications, and outcome of patients with angiogram-negative SAH.

**Methods:**

We retrospectively reviewed all adult patients admitted to a neurosurgical intensive care unit during 2004–2018 due to spontaneous angiogram-negative SAH. Our primary outcome was a dichotomized Glasgow Outcome Scale (GOS) at 3 months. We assessed factors that associated with outcome using multivariable logistic regression analysis.

**Results:**

Of the 108 patients included, 84% had a favorable outcome (GOS 4–5), and mortality was 5% within 1 year. The median age was 58 years, 51% were female, and 93% had a low-grade SAH (World Federation of Neurosurgical Societies grading I–III). The median number of angiograms performed per patient was two. Thirty percent of patients showed radiological signs of acute hydrocephalus, 28% were acutely treated with an external ventricular drain, 13% received active vasospasm treatment and 17% received a permanent shunt. In the multivariable logistic regression model, only acute hydrocephalus associated with unfavorable outcome (odds ratio = 4.05, 95% confidence interval = 1.05–15.73). Two patients had a new bleeding episode.

**Conclusion:**

SAH-related complications such as hydrocephalus and vasospasm are common after angiogram-negative SAH. Still, most patients had a favorable outcome. Only acute hydrocephalus was associated with unfavorable outcome. The high rate of SAH-related complications highlights the need for neurosurgical care in these patients.

**Supplementary Information:**

The online version contains supplementary material available at 10.1007/s00701-021-05069-7.

## Introduction

Spontaneous subarachnoid hemorrhage (SAH) causes 2–9% of all strokes, but it is one of the most common causes of stroke-related deaths among the middle-aged [22, 25]. Ruptured intracranial aneurysms are the most common cause of spontaneous SAH (85%). In approximately two-thirds of the remaining (10%), the bleeding source cannot be identified; these are referred as spontaneous angiogram-negative SAH; the residual (5%) are due to various rare conditions consisting mainly of arteriovenous malformations [27]. These patients can be divided into two distinct categories based upon the distribution pattern of SAH. These include the perimesencephalic pattern (PMH) with center of the blood pooled in cisterns around the midbrain or the diffuse pattern (nPMH) [23, 24]. Typically, both have a benign clinical course compared to aneurysmal SAH (aSAH) with fewer complications such as hydrocephalus, vasospasm, and delayed cerebral ischemia (DCI) [23, 27]. Furthermore, the prognosis of PMH and nPMH is considered good, with 83–100% recovering with a favorable (4–5) Glasgow Outcome Score (GOS) compared to 59% of those with aSAH [5, 8, 9, 26].

Few studies have looked at larger cohorts of patients with angiogram-negative SAH. A study by Little et al. [16] found those with nPMH to have worse outcome compared to PMH, a 25% risk of angiographic vasospasm and a 6% risk for long-term hydrocephalus. Notably, is that 6–8% of patients have been reported to experience re-hemorrhage as a long-term complication after angiogram-negative SAH [3, 23] .

The aim with this retrospective cohort study is to describe the occurrence, clinical course, and outcome of patients with spontaneous angiogram-negative SAH treated in a large academic non-profit university hospital.

## Methods

### Ethics

The study was approved by the research committee of Helsinki University Hospital. Due to the retrospective study nature, no informed consent was obtained.

### Study setting

We retrospectively reviewed all patients with angiogram-negative SAH admitted to the neurosurgical care unit (neuro-ICU) of Helsinki University Hospital from September 2004 to January 2018. Helsinki University Hospital is the largest university hospital in Finland, with a catchment area of approximately 2 million people (one third of the population in Finland). The treatment of SAH has been centralized to the neurosurgical department for decades.

### Patient population

We screened all adult patients (≥ 18 years) with an International Statistical Classification of Diseases and Health Problems, tenth revision (ICD-10) code of I60.7, I60.8, or I60.9 to the designated neuro-ICU. These patients’ electronic healthcare records and radiological images were scrutinized. We considered only those with negative angiographic studies (including computed tomography angiogram, magnetic resonance angiogram, and digital subtraction angiogram). If the patient had an angiographic study, showing an intracranial aneurysm or other visible bleeding etiology the patient was not included. A minimum of one digital subtraction angiogram (DSA), computerized tomography angiogram (CTA), or magnetic resonance angiogram (MRA) examination was needed to rule out a ruptured aneurysm or other malformation. Patients with a history of trauma prior to the illness/SAH were excluded, as were patients that were found to have vasculitis or reversible cerebral vasoconstriction syndrome.

At our institution, all conscious patients with SAH received per oral nimodipine and tranexamic acid (unconscious patients received intravenous nimodipine). Tranexamic acid was continued for 72 hours or until an aneurysmal bleeding source was excluded (after first or second angiography). After exclusion of aneurysmatic SAH, nimodipine was continued for 5 to 7 days.

### Data collection and definition of variables

All data were collected through electronic healthcare records and radiological images. The data collected included patient characteristics, time of symptom onset, smoking habits, hypertension, antithrombotic medication, and number and type of performed angiograms together with length of hospital and ICU stay. Pupillary light reflexes, focal neurological symptoms, Glasgow Coma Scale (GCS) scores, the World Federation of Neurosurgical Societies (WFNS) grading scale, and Hunt and Hess (H&H) scores were evaluated upon admission to the emergency department (ED) or preceding sedation and intubation. A WFNS score of 1–3 was considered low grade, and a score of 4–5 was considered high grade. Acute hydrocephalus was defined as an acute radiological enlargement of the brain ventricles, with or without treatment. The modified Fisher scale was based upon the admission imaging as were radiological appearance of a typical or atypical PMH.

Treatment-related variables such as hydrocephalus demanding external ventricular drainage (EVD), spinal drainage, or cerebrospinal fluid (CSF) shunt were collected.

Radiological vasospasm and delayed cerebral ischemia (DCI) were registered. DCI was defined as if a patient developed new neurological deficits not attributable to a previous focal lesion with or without radiological vasospasm. Actively treated vasospasm or DCI was registered. Generally, vasospasm treatment was initiated in case of DCI. Radiological vasospasm without clinical findings was not a treatment trigger. Yet, if a reliable neurological assessment was not possible, treatment was initiated in case of severe radiological vasospasm. Routine angiographic studies to diagnose or rule out radiological vasospasm were not performed.

### Outcomes

Our primary outcome of interest was the Glasgow Outcome Score (GOS) 3 months after admission. The GOS was categorized into favorable (GOS 4–5) and unfavorable (GOS ≤ 3) outcome. Additionally, rebleeding rates (assessed August 1, 2019) and 1-year case fatality are described as well.

### Statistics

Continuous variables were examined for distribution. Normally distributed data were analyzed using an independent *t* test, whereas non-parametric data were analyzed with a non-parametric Mann-Whitney *U* test. Categorical variables were compared between groups using a χ^2^ test (Fisher’s exact test when needed). To assess the association between predictors and outcome, we first compared variables in univariate analysis. We considered *p* values < 0.05 as statistically significant. Significant not treatment-related variables that associated with outcome in the univariate analysis were included in a multivariable logistic regression model to find independent predictors associated with outcome. To avoid collinearity, we included WFNS grade instead of GCS score as WFNS grade is established in the SAH literature and allows for dichotomization into poor (WFNS IV to V) and good grade (WFNS I to III) [8]. We did not include variables from the univariate analysis with a high number of missing values. Age was included in all multivariable analyses. We separately analyzed patients with PMH and nPMH. SPSS Statistics version 25.0 for Windows (IBM Corp, Armonk, NY, USA) was used for all statistical analyses.

## Results

**Fig. 1.**
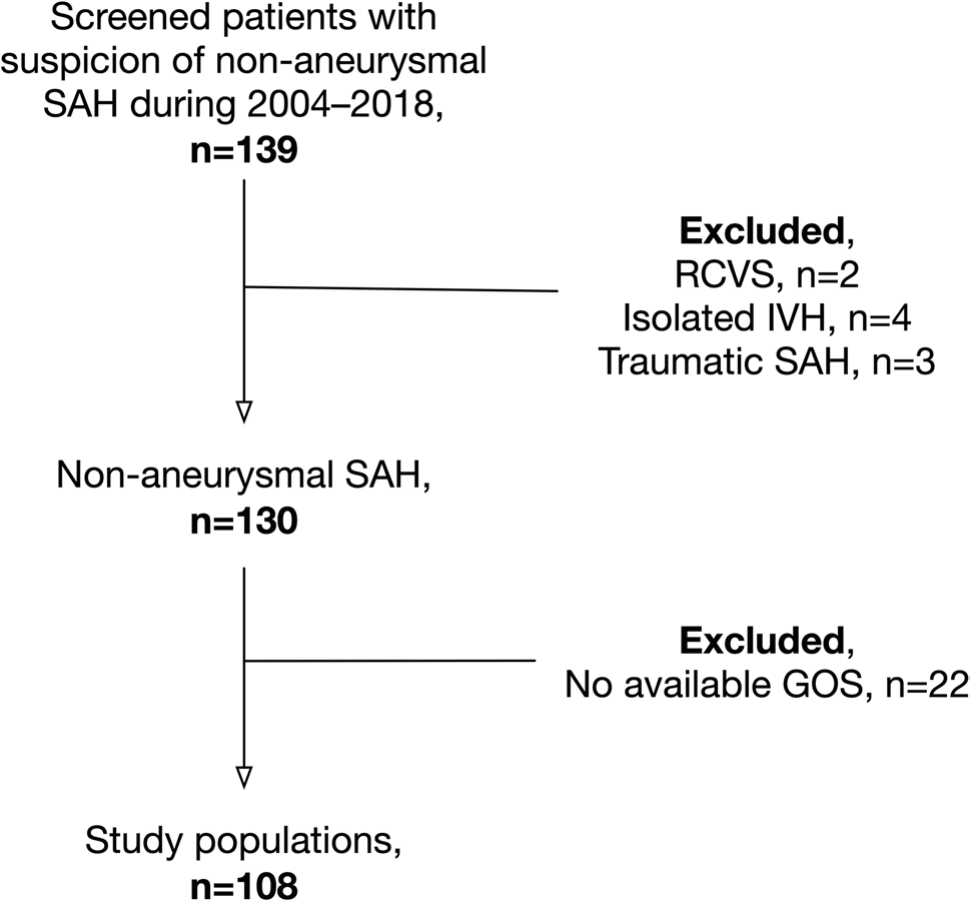
Flow chart showing study population

Of 139 screened patients, a total of 130 patients met the inclusion criteria (Fig. 1). All SAH was diagnosed with a CT scan, except for two patients who had a normal CT scan and whose SAH was diagnosed with lumbar puncture. Of the 130 patients, 22 patients did not have any follow-up data. Thus, a total of 108 patients had compete follow-up data. There were no major differences in patient characteristics and treatment-related variables between patients with follow-up data and without follow-up data (Digital Supplemental 1).

Patient characteristics are shown in Table 1. The median age was 58 years, and approximately half were female. Fifteen percent were on some form of antithrombotic medication, and 36% had a previous diagnosis of hypertension. The majority of patients (69%) had a GCS score of 15 on admission. Seven patients (7%) were considered having high-grade SAH. Almost three out of four had a modified Fisher grade of 0 to 2, 54% had intraventricular hemorrhage (IVH), 30% had radiological and clinical signs of acute hydrocephalus, and 37% had a typical PMH distribution.

**Table 1:** Patient characteristics and treatment-related variables

Variable	Patients (*n* = 108)
Age, median (IQR)	58 (50–66)
Sex	
Female	55 (51%)
Male	53 (49%)
Time from symptom onset to admission (days), median (IQR)	0 (0–1)
Smoking	
Yes	15 (14%)
No	33 (31%)
Ex-smoker	8 (7%)
Unknown	52 (48%)
Antithrombotic medication	16 (15%)
Anticoagulation	6 (6%)
Antiplatelet	8 (7%)
Both	2 (2%)
No	92 (85%)
Hypertension	39 (36%)
GCS score	
15	75 (69%)
13–14	23 (21%)
7–12	7 (7%)
< 7	3 (3%)
Pupillary light reactivity	
Normal	105 (97%)
Abnormal	3 (3%)
Focal neurological symptom	8 (7%)
WFNS grade	
1–3	101 (93%)
4–5	7 (7%)
Radiological variables	
Modified Fischer grade	
0	1 (1%)
1	37 (34%)
2	36 (33%)
3	12 (11%)
4	22 (21%)
IVH	58 (54%)
Lateral ventricles	30 (28%)
III ventricle	23 (21%)
IV ventricle	48 (44%)
Typical PMH distribution*	40 (37%)
Acute hydrocephalus	32 (30%)
Treatment-related variables	
External ventricular drain	30 (28%)
Time of drainage (days), median (IQR) †	8 (5–14)
Spinal drainage	17 (16%)
Time of drainage (days), median (IQR)‡	7 (4–10)
CSF shunt	18 (17%)
Days from admission to shunt, median (IQR)	26 (14–40)
Radiological vasospasm	17 (16%)
Clinical DCI	6 (6%)
Active DCI/vasospasm treatment	14 (13%)
ICU length of stay (days), median (IQR)	2 (1–7)
Hospital length of stay (days), median (IQR)	9 (6–14)

Abbreviations: *CSF* cerebrospinal fluid; *DCI* Delayed Cerebral Ischemia; *GCS* Glasgow Coma Scale; *ICH* intracerebral hemorrhage; *ICU* intensive care unit; *IQR* interquartile range; *IVH* intraventricular hemorrhage; *PMH* perimesencephalic subarachnoid hemorrhage; *WFNS* World Federation of Neurosurgical Societies

*missing for 1 patient

†missing for 4 patients

‡missing for 3 patients

Of those with acute hydrocephalus, 56% needed acute CSF diversion and 44% had a ventriculoatrial or ventriculoperitoneal shunt.

### Angiograms

A total of 210 angiographic studies were performed on all patients. Of these 78% were CTA, 5% MRA, and 17% DSA. The median number of angiograms performed per patient was 2 (IQR 1–2). Sixty-three percent of all patients required a repeat angiogram, 24% of all patients underwent three or more angiograms, and 28% of all patients had at least one DSA performed.

### Treatment-related variables

Thirty-nine patients (36%) required some sort of CSF drainage. Of these, 30 patients had an EVD, 17 patients had a spinal drainage, and 18 patients received a ventriculoperitoneal or ventriculoatrial shunt (one patient could have one or several procedures). Median EVD treatment time was 8 days (IQR 5–14), and median spinal drainage time was 7 days (IQR 4-10). Median time from admission to shunting was 26 days (IQR 14–40). DCI was registered in 6% of patients. Isolated radiological vasospasm was registered in 16% of patients. Thirteen percent of patients received active vasospasm treatment.

### Patient outcomes

The median time to GOS assessment was 82 days (IQR 52–105). The majority of patients (84%) had a favorable functional outcome (GOS 4–5). Median length of stay in the hospital was 9 days (IQR 6-14), and median length of stay in the neuro-ICU was 2 days (IQR 1-7). Five patients (5%) died within 1 year from admission. Only one patient died during the index hospitalization, 6 days after admission (massive SAH with hydrocephalus, IVH, and brain infarction). One of the deceased died due to diabetic ketoacidosis and infection during the convalescence 111 days later, the other patients died primarily from SAH related causes (Digital Supplemental 2).

### Re-bleedings

Median rebleeding follow-up time was 7.6 years (IQR 3.3–11.4 years). One patient suffered a re-bleed 6 days after the initial SAH. For another patient, this hospitalization was a re-bleed episode from a previous spontaneous angiogram-negative SAH. These patients are more thoroughly described in the Digital Supplemental 2.

### Multivariable analysis

Differences in patient characteristics between those with a favorable and unfavorable outcome are shown in Table 2. A total of 107 patients were included in the final multivariable logistic regression analysis (Table 3). Of the included variables, only acute hydrocephalus (odds ratio, OR 4.93; 95% confidence interval, CI 1.14–21.41, *p* = 0.033) showed an independent association with an unfavorable outcome.

**Table 2:** Patient characteristics and treatment-related variables according to functional outcome

Variable	Favorable outcome (*N* = 91)	Unfavorable outcome (*N* = 17)	*p value*
Age, median (IQR)	58 (49–64)	66 (54–72)	0.045
Sex			
Female	47 (52%)	8 (47%)	0.795
Male	44 (48%)	9 (53%)	
Time from symptom onset to admission (days), median (IQR)	0 (0–1)	0 (0–1)	0.737
Smoking			
Yes	14 (15%)	1 (6%)	0.014
No	32 (35%)	1 (6%)	
Ex-smoker	7 (8%)	1 (6%)	
Unknown	38 (42%)	14 (82%)	
Antithrombotic medication	10 (11%)	6 (35%)	0.010
Anticoagulation	2 (2%)	4 (24%)	
Antiplatelet	6 (7%)	2 (12%)	
Both	2 (2%)	0 (0%)	
No	81 (89%)	11 (65%)	
Hypertension	32 (35%)	7 (41%)	0.784
GCS score			
15	72 (79%)	3 (18%)	< 0.001
13–14	16 (18%)	7 (41%)	
7–12	2 (2%)	5 (29%)	
< 7	1 (1%)	2 (12%)	
Pupillary light reactivity			
Normal	90 (99%)	15 (88%)	0.064
Abnormal	1 (1%)	2 (12%)	
Focal neurological symptom	5 (6%)	3 (18%)	0.110
WFNS grade			
1–3	88 (97%)	13 (77%)	0.011
4–5	3 (3%)	4 (23%)	
Radiological variables			
Modified Fisher grade			
0	1 (1%)	0 (0%)	0.008
1	34 (37%)	3 (18%)	
2	31 (34%)	5 (29%)	
3	12 (13%)	0 (0%)	
4	13 (14%)	9 (53%)	
Modified Fisher grades 0–2	66 (73%)	8 (47%)	0.049
Modified Fisher grades 3–4	25 (28%)	9 (53%)	
IVH	44 (49%)	14 (82%)	0.015
Lateral ventricles	17 (19%)	13 (77%)	< 0.001
III ventricle	19 (21%)	4 (24%)	0.756
IV ventricle	37 (41%)	11 (65%)	0.109
Typical PMH distribution*	38 (42%)	2 (13%)	0.027
Acute hydrocephalus	21 (23%)	11 (65%)	0.001
Treatment-related variables			
External ventricular drain	17 (19%)	13 (77%)	< 0.001
Time of drainage (days), median (IQR)†	7 (4–10)	11 (7–17)	0.087
Spinal drainage	13 (14%)	4 (24%)	0.456
Time of drainage (days), median (IQR)‡	6 (3–7)	11 (7–13)	0.054
CSF shunt	10 (11%)	8 (47%)	0.001
Days from admission to shunt, median (IQR)	25 (4–52)	28 (19–33)	0.696
Radiological vasospasm	13 (14%)	4 (24%)	0.466
Clinical DCI	5 (6%)	1 (6%)	1.000
Active DCI/vasospasm treatment	10 (11%)	4 (24%)	0.229
ICU length of stay (days), median (IQR)	2 (1–4)	10 (3–19)	< 0.001
Hospital length of stay (days), median (IQR)	9 (6–13)	14 (5–32)	0.088

Favorable outcome is defined as a Glasgow Outcome Scale of 4–5, and unfavorable outcome is defined as a Glasgow Outcome Scale of 1–3.

Abbreviations: *CSF* cerebrospinal fluid; *DCI* delayed cerebral ischemia; *GCS* Glasgow Coma Scale; *ICH* intracerebral hemorrhage; *ICU* intensive care unit; *IQR* interquartile range; *IVH* intraventricular hemorrhage; *PMH* perimesencephalic subarachnoid hemorrhage; *WFNS* World Federation of Neurosurgical Societies

*missing for 1 patient

†missing for 4 patients

‡missing for 3 patients

**Table 3:** Multivariable logistic regression model showing predictors of poor functional outcome

Variable	OR (95% CI)	*p* value
Age (cont.)	1.05 (0.99–1.12)	0.106
Antithrombotic medication		
No	Ref	
Yes	2.90 (0.64–13.05)	0.166
WFNS SAH grade		
1–3	Ref	
4–5	2.93 (0.42–20.66)	0.281
Modified Fisher grade		
0–2	Ref	
3–4	3.16 (0.68–14.57)	0.141
IVH		
No	Ref	
Yes	2.78 (0.64–12.08)	0.173
Acute hydrocephalus		
No	Ref	
Yes	4.93 (1.14–21.41)	0.033
Typical PMH		
No	1.14 (0.16–8.23)	0.897
Yes	Ref	

Favorable outcome is defined as a Glasgow Outcome Scale of 4–5, and unfavorable outcome is defined as a Glasgow Outcome Scale of 1–3.

Abbreviations: *CI* confidence interval*; Cont. continuous; IVH* intraventricular hemorrhage; *OR* odds ratio*; Ref reference; SAH* subarachnoid hemorrhage*; PMH* perimesencephalic subarachnoid hemorrhage; *WFNS* World Federation of Neurosurgical Societies

### PMH versus nPMH

Differences between patients with favorable and unfavorable outcomes in the PMH and nPMH groups are shown in Table 4. In the PMH group, there were only two patients with unfavorable outcome, and, thus, a more detailed analysis regarding risk factors for unfavorable outcome was not possible.

**Table 4:** Patient characteristics and treatment-related variables according to functional outcome for patients with perimesencephalic subarachnoid hemorrhage (PMH) and diffuse pattern perimesencephalic subarachnoid hemorrhage (nPMH)

Variable	PMH (*n* =40)			nPMH (*n* = 67)		
	Favorable outcome (*N* = 38)	Unfavorable outcome (*N* = 2)	*p value*	Favorable outcome (*N* = 53)	Unfavorable outcome (*N* = 14)	*p value*
Age, median (IQR)	57 (49–62)	72 (71–72)	0.005	58 (50–68)	62 (51–69)	0.468
Sex						
Female	21 (55%)	1 (50%)	1.000	26 (49%)	7 (50%)	1.000
Male	17 (45%)	1 (50%)		27 (51%)	7 (50%)	
Time from symptom onset to admission (days), median (IQR)	0 (0–1)	1 (0–1)	0.785	0 (0–1)	0 (0–1)	0.735
Smoking						
Yes	8 (21%)	0 (0%)	0.272	6 (11%)	1 (7%)	0.157
No	15 (40%)	0 (0%)		17 (32%)	1 (7%)	
Ex-smoker	5 (13%)	1 (50%)		2 (4%)	0 (0%)	
Unknown	10 (26%)	1 (50%)		28 (53%)	12 (86%)	
Antithrombotic medication	35 (92%)	1 (50%)	0.192	7 (13%)	5 (36%)	0.109
Anticoagulation	0 (0%)	0 (0%)		2 (4%)	4 (29%)	0.039
Antiplatelet	3 (8%)	1 (50%)		3 (6%)	1 (7%)	
Both	0 (0%)	0 (0%)		2 (4%)	0 (0%)	
No	35 (92%)	1 (50%)		46 (87%)	9 (64%)	
Hypertension	12 (32%)	1 (50%)	1.000	20 (38%)	6 (43%)	0.765
GCS score						
15	30 (79%)	1 (50%)	0.086	42 (79%)	2 (14%)	< 0.001
13–14	8 (21%)	0 (0%)		8 (15%)	6 (43%)	
7–12	0 (0%)	1 (50%)		2 (4%)	4 (29%)	
< 7	0 (0%)	0 (0%)		1 (2%)	2 (14%)	
Pupillary light reactivity						
Normal	38 (100%)	2 (100%)		52 (98%)	12 (86%)	0.108
Abnormal	0 (0%)	0 (0%)		1 (2%)	2 (14%)	
Focal neurological symptom	2 (5%)	0 (0%)	1.000	3 (6%)	3 (21%)	0.100
WFNS grade						
1–3	38 (100%)	2 (100%)		50 (94%)	10 (71%)	0.030
4–5	0 (0%)	0 (0%)		3 (6%)	4 (29%)	
Radiological variables						
Modified Fisher grade						
0	0 (0%)	0 (0%)	1.000	1 (2%)	0 (0%)	0.047
1	21 (55%)	1 (50%)		13 (25%)	2 (14%)	
2	17 (45%)	1 (50%)		14 (26%)	3 (21%)	
3	0 (0%)	0 (0%)		12 (23%)	0 (0%)	
4	0 (0%)	0 (0%)		13 (24%)	9 (64%)	
Modified Fisher grades 0–2	38 (100%)	2 (100%)		28 (53%)	5 (36%)	0.369
Modified Fisher grades 3–4	0 (0%)	0 (0%)		25 (47%)	9 (64%)	
IVH	17 (45%)	1 (50%)	1.000	27 (51%)	12 (86%)	0.031
Lateral ventricles	3 (8%)	1 (50%)	0.192	14 (26%)	11 (79%)	< 0.001
III ventricle	6 (16%)	0 (0%)	1.000	13 (25%)	4 (29%)	0.740
IV ventricle	15 (40%)	0 (0%)	0.519	22 (42%)	10 (71%)	0.071
Acute hydrocephalus	5 (13%)	0 (0%)	1.000	16 (30%)	11 (79%)	0.002
Treatment-related variables						
External ventricular drain	4 (11%)	1 (50%)	0.237	13 (25%)	11 (79%)	< 0.001
Time of drainage (days), median (IQR)	7 (6–14)	NA		6 (3–10)	10 (6–18)	0.169
Spinal drainage	4 (11%)	0 (0%)	1.000	9 (17%)	4 (29%)	0.447
Time of drainage† (days), median (IQR)	6 (6)†			4 (2–8)	11 (7–13)	0.042
CSF shunt	1 (3%)	1 (50%)	0.099	9 (17%)	7 (50%)	0.029
Days from admission to shunt, median (IQR)	2	34	1.000	26 (10–55)	26 (18–30)	1.000
Radiological vasospasm	5 (13%)	1 (50%)	0.281	8 (15%)	3 (21%)	0.686
Clinical DCI	2 (5%)	0 (0%)	1.000	3 (6%)	1 (7%)	1.000
Active DCI/vasospasm treatment	3 (8%)	1 (50%)	0.192	7 (13%)	3 (21%)	0.425
ICU length of stay (days), median (IQR)	1 (1–2)	10 (3–19)	0.974	2 (1–6)	12 (6–20)	< 0.001
Hospital length of stay (days), median (IQR)	9 (5–11)	2 (1–2)	0.077	10 (6–15)	25 (8–36)	0.017

Favorable outcome is defined as a Glasgow Outcome Scale of 4–5, and unfavorable outcome is defined as a Glasgow Outcome Scale of 1–3.

Abbreviations: *CSF* cerebrospinal fluid; *DCI* delayed cerebral ischemia; *GCS* Glasgow Coma Scale; *ICH* intracerebral hemorrhage; *ICU* intensive care unit; *IQR* interquartile range; *IVH* intraventricular hemorrhage; *PMH* perimesencephalic subarachnoid hemorrhage; *WFNS* World Federation of Neurosurgical Societies

†missing for 3 patient

For patients with nPMH, those with an unfavorable outcome had higher WFNS grade, higher modified Fisher grade, more frequent IVH, and more frequent acute hydrocephalus. In a multivariable logistic regression model including these and age, only acute hydrocephalus remained associated with unfavorable outcome (OR 8.56, 95% CI 1.59–46.23, Table 5).

**Table 5:** Multivariable logistic regression model showing predictors of poor functional outcome in patients with diffuse pattern perimesencephalic subarachnoid hemorrhage

Variable	OR (95% CI)	*p* value
Age (cont.)	1.03 (0.97–1.10)	0.338
WFNS SAH grade		
1–3	Ref	
4–5	2.74 (0.41–18.47)	0.299
Modified Fisher grade		
0–2	Ref	
3–4	3.08 (0.67–14.17)	0.148
IVH		
No	Ref	
Yes	4.47 (0.76–26.27)	0.098
Acute hydrocephalus		
No	Ref	
Yes	8.56 (1.59–46.23)	0.013

Favorable outcome is defined as a Glasgow Outcome Scale of 4–5, and unfavorable outcome is defined as a Glasgow Outcome Scale of 1–3.

Abbreviations: *CI* confidence interval*; Cont.* continuous*; IVH* intraventricular hemorrhage*; OR* odds ratio*; Ref reference; SAH* subarachnoid hemorrhage*; nPMH diffuse pattern perimesencephalic subarachnoid hemorrhage; WFNS* World Federation of Neurosurgical Societies

Additionally, we compared patient characteristics between the PMH and nPMH group (Table 6). Patients with nPMH were more likely to have higher WFNS and modified Fisher grade, IVH in the lateral ventricles, acute hydrocephalus, permanent CSF shunt, and longer ICU and hospital stay.

**Table 6:** Differences in patient characteristics and treatment-related variables for patients with perimesencephalic subarachnoid hemorrhage (PMH) and diffuse pattern Perimesencephalic Subarachnoid hemorrhage (nPMH)

Variable	PMH (*N* = 40)	nPMH (*N* = 67)	*p value*
Age, median (IQR)	58 (49–63)	59 (50–67)	0.230
Sex			
Female	22 (55%)	33 (49%)	0.565
Male	18 (45%)	34 (51%)	
Time from symptom onset to admission (days), median (IQR)	0 (0–1)	0 (0–1)	0.823
Smoking			
Yes	8 (20%)	7 (10%)	0.004
No	15 (38%)	18 (27%)	
Ex-smoker	6 (15%)	2 (3%)	
Unknown	11 (27%)	40 (60%)	
Antithrombotic medication	4 (10%)	12 (18%)	0.402
Anticoagulation	0 (0%)	6 (9%)	
Antiplatelet	4 (10%)	4 (6%)	
Both	0 (0%)	2 (3%)	
No	36 (90%)	55 (82%)	
Hypertension	13 (33%)	26 (39%)	0.512
GCS score			
15	31 (78%)	44 (66%)	0.345
13–14	8 (20%)	14 (21%)	
7–12	1 (2%)	6 (9%)	
< 7	0 (0%)	3 (4%)	
Pupillary light reactivity			
Normal	40 (100%)	64 (96%)	0.291
Abnormal	0 (0%)	3 (4%)	
Focal neurological symptom	2 (5%)	6 (9%)	0.707
WFNS grade			
1–3	40 (100%)	60 (90%)	0.044
4–5	0 (0%)	7 (10%)	
Radiological variables			
Modified Fisher grade			
0	0 (0%)	1 (2%)	< 0.001
1	22 (55%)	15 (22%)	
2	18 (45%)	17 (25%)	
3	0 (0%)	12 (18%)	
4	0 (0%)	22 (33%)	
Modified Fisher grades 0–2	40 (100%)	33 (49%)	< 0.001
Modified Fisher grades 3–4	0 (0%)	34 (51%)	
IVH	18 (45%)	39 (58%)	0.185
Lateral ventricles	4 (10%)	25 (37%)	0.002
III ventricle	6 (15%)	17 (25%)	0.234
IV ventricle	15 (38%)	32 (48%)	0.301
Acute hydrocephalus	5 (13%)	27 (40%)	0.002
Treatment-related variables			
External ventricular drain	5 (13%)	24 (36%)	0.009
Time of drainage (days), median (IQR)*	7 (6–14)	8 (5–13)	0.971
Spinal drainage	4 (10%)	13 (19%)	0.277
Time of drainage (days), median (IQR)	6 (6)	7 (3–11)	0.885
CSF shunt	2 (5%)	16 (24%)	0.015
Days from admission to shunt, median (IQR)	18 (2–18)	26 (16–47)	0.641
Radiological vasospasm	6 (15%)	11 (16%)	1.000
Clinical DCI	2 (5%)	4 (6%)	1.000
Active DCI/vasospasm treatment	4 (10%)	10 (15%)	0.563
ICU length of stay (days), median (IQR)	1 (1–2)	2 (1–10)	0.016
Hospital length of stay (days), median (IQR)	8 (4–11)	10 (6–17)	0.044

Favorable outcome is defined as a Glasgow Outcome Scale of 4–5, and unfavorable outcome is defined as a Glasgow Outcome Scale of 1–3.

Abbreviations: *CSF* cerebrospinal fluid; *DCI* delayed cerebral Ischemia; *GCS* Glasgow Coma Scale; *ICH* intracerebral hemorrhage; *ICU* intensive care unit; *IQR* interquartile range; *IVH* intraventricular hemorrhage; *nPMH* diffuse pattern perimesencephalic subarachnoid hemorrhage; *PMH* perimesencephalic subarachnoid hemorrhage; *WFNS* World Federation of Neurosurgical Societies

*missing for 4 patients

†missing for 3 patients

## Discussion

### Key findings

We investigated the clinical course, SAH-related complications, and outcome of patients with angiogram-negative SAH over a 14-year period. We found that the vast majority of patients had a favorable neurological outcome. Still, 5% of patients died within one-year of admission. SAH-related complications, such as hydrocephalus and vasospasm, were common but re-hemorrhage was rare.

We found greater age, smoking, antithrombotic medication, low GCS and WFNS upon administration, modified Fisher, presence of IVH, atypical PMH distribution, acute hydrocephalus, need of CSF diversion, and radiological vasospasm to associate with unfavorable outcome in univariate analysis. However, in multivariable logistic regression analysis, only acute hydrocephalus showed an independent association with unfavorable outcome.

### Comparison with previous studies

Unfavorable outcome occurred in 16% of our patients, which is similar to previous studies [9, 14, 16, 20]. Konczalla et al. found that 83% had an favorable neurological outcome, defined as modified Rankin Scale grading 0–2, in a cohort similar to our findings [14]. However, the all-cause mortality of 5% in our findings appears to be slightly higher compared to others. One study reported a mortality rate of 4% during a 45 month follow-up period [23], of which half died primarily from cancer, while another study reported a mortality rate as high as 10%, 6 months after admission [14]. Our rebleeding risk of 2% (2 patients) matches the previous numbers of 0–4% reported in previous studies after a follow-up ranging 0.8–90 months [3, 13, 18, 20, 23].

In the present study, only acute hydrocephalus was associated with unfavorable outcome. Acute hydrocephalus has also been described to associate with unfavorable outcome in other settings [14]. In comparison to previous studies, we did not find high grade SAH (defined as a WFNS of 4–5) to be associated with unfavorable outcome [14]. This is probably due to the low number of high grade SAH patients in our cohort (7 patients). Although it did not reach statistical significance, higher age seems to associate with a higher probability of unfavorable outcome.

Almost a third (30%) of our patients displayed radiological signs of acute hydrocephalus. In comparison, approximately 40%, of patients with aneurysmal SAH, show radiological signs of acute hydrocephalus on admission [6]. Our number seems rather high but is comparable to other series of angiogram-negative SAH patients (11–29%) [9, 11, 13, 14, 20]. Reasons for the high occurrence of acute hydrocephalus are probably the high occurrence of IVH (54%). Acute hydrocephalus is also associated with poorer outcome in aneurysmal SAH [5, 28]. Hydrocephalus associated poor outcome after SAH is probably related to a decrease in cerebral blood perfusion [26], stretching and damaging of the ventricle walls [19], and the corpus callosum [17].

Sixteen percent of our patients displayed signs of radiological vasospasm at some point during the hospitalization, while 13% received active vasospasm treatment. This number is lower than the occurrence of vasospasm in patients with aneurysmal SAH (33%) [2, 12, 21]. Also, previous studies with angiogram-negative SAH patients have showed similar rates of vasospasm (19%) [15]. Still, whether the pathophysiology leading to vasospasm and delayed cerebral ischemia after angiogram-negative SAH and aneurysmal SAH are similar remains unknown.

The etiology of spontaneous angiogram-negative SAH has yet to be established. It is thought to originate from a venous origin, given its amount of hemorrhage and benign clinical course [23]. A report by Hafez et al. found a venous saccular aneurysm in the lateral pontine vein, in a patient with PMH [7]. Another theory suggests a leakage from the ventriculostriate and thalamoperforating vessels [1]. Modern imaging methods enable more profound investigations of the arterial and venous systems that could help determine the origin for angiogram-negative SAH.

### Strengths and limitations

There are some strengths that need to be highlighted. Because the care of SAH patients has been centralized to our department for decades, our cohort is comprehensive. Although there probably are angiogram-negative SAH patients never seeking hospital care, our study sample is representative of the situation in our catchment area during the study time. Further, healthcare records have stored electronically since the early 2000s, making the data readily available and reliable.

However, some limitations should be mentioned. Our study is retrospective and our cohort stems from one center. Thus, generalizability of our results to other settings might be limited. All patients did not undergo DSA. At our institution, primary screening is done with CTA. Invasive DSA is mainly done to patients with a disproportionate amount of SAH or to patients in poor clinical condition. We had no structured follow-up of patients with angiogram-negative SAH, causing a 15% of lacking outcome data. Also, our cohort comes from a tertiary neuro-ICU. It is possible that some angiogram-negative SAH patients never seek medical care and thus, our, reported numbers (for example, 30% acute hydrocephalus) are overestimating the risk of SAH-related complications. Further, we assessed outcome according to GOS, which does not regard the patient’s neuropsychological state or perceived quality of life. The neuropsychological component has been previously reported as an issue in patients with angiogram-negative SAH, and it would be an implication for further research [4].

## Conclusions

Although angiogram-negative SAH is considered a benign illness, we found rather high rates of SAH-related complications. Still, the vast majority did recover to a favorable outcome. Only acute hydrocephalus was associated with unfavorable outcome. The high rate of SAH-related complications highlights the need for neurosurgical care in these patients.

## Supplementary Information

ESM 1 (DOCX 16 kb)

ESM 2 (DOCX 711 kb)
