# Supplementary material for: Spontaneous angiogram-negative subarachnoid hemorrhage: a retrospective single center cohort study
Source: Acta Neurochir (Wien). 2021 Dec 1;164(1):129–40. doi: 10.1007/s00701-021-05069-7 (PMC8761132; doi:10.1007/s00701-021-05069-7)
Supplement: Supplementary file 1 — (DOCX 16 kb) [file 701_2021_5069_MOESM1_ESM.docx]

| **Digital Supplementary 1**: Comparison of patient characteristics and treatment-related variables for patients excluded due to missing follow-up data with included patients | | |
| --- | --- | --- |
| **Variable** | **Patients excluded (n=22)** | **Patients included (n=108)** |
| Age, median (IQR) | 54 (54-70) | 58 (50-66) |
| Sex |  |  |
| Female | 11 (50%) | 55 (51%) |
| Male | 11 (50%) | 53 (49%) |
| Time from symptom onset to admission (days), median (IQR) | 0 (0-1) | 0 (0-1) |
| Smoking |  |  |
| Yes | 3 (14%) | 15 (14%) |
| No | 12 (54%) | 33 (31%) |
| Ex-smoker | 0 (0%) | 8 (7%) |
| Unknown | 7 (32%) | 52 (48%) |
| Antithrombotic medication |  |  |
| Anticoagulation | 1 (5%) | 6 (6%) |
| Antiplatelet | 2 (9%) | 8 (7%) |
| Both | 0 (0%) | 2 (2%) |
| No | 19 (86%) | 92 (85%) |
| Hypertension | 9 (41%) | 39 (36%) |
| GCS score |  |  |
| 15 | 18 (82%) | 75 (69%) |
| 13-14 | 3 (14%) | 23 (21%) |
| 7-12 | 1 (5%) | 7 (7%) |
| <7 | 0 (0%) | 3 (3%) |
| Pupillary light reactivity |  |  |
| Normal | 22 (100%%) | 105 (97%) |
| Abnormal | 0 (0%) | 3 (3%) |
| Focal neurological symptom | 2 (9%) | 8 (7%) |
| WFNS grade |  |  |
| 1-3 | 21 (95%) | 101 (93%) |
| 4-5 | 1 (5%) | 7 (7%) |
| **Radiological variables** |  |  |
| modified Fischer grade |  |  |
| 0 | 1 (5%) | 1 (1%) |
| 1 | 8 (36%) | 37 (34%) |
| 2 | 9 (41%) | 36 (33%) |
| 3 | 2 (9%) | 12 (11%) |
| 4 | 2 (9%) | 22 (21%) |
| ICH | 1 (5%) | 0 (0%) |
| IVH | 11 (50%) | 58 (54%) |
| Lateral ventricles | 7 (32%) | 30 (28%) |
| III-ventricle | 3 (14%) | 23 (21%) |
| IV-ventricle | 8 (36%) | 48 (44%) |
| Typical PMH distribution | 7 (32%) | 40 (37%) |
| Acute hydrocephalus | 3 (14%) | 32 (30%) |
| **Treatment related variables** |  |  |
| External ventricular drain | 3 (14%) | 30 (28%) |
| Time of drainage (days), median (IQR) | 2 (2-3) | 8 (5-14) |
| Spinal drainage | 1 (5%) | 17 (16%) |
| Time of drainage (days), median (IQR) | 6* | 7 (4-10) |
| CSF shunt | 0 (0%) | 18 (17%) |
| Days from admission to shunt, median (IQR) |  | 26 (14-40) |
| Radiological vasospasm | 0 (0%) | 17 (16%) |
| Clinical DCI | 0 (0%) | 6 (6%) |
| Active DCI/vasospasm treatment | 0 (0%) | 14 (13%) |
| ICU length of stay (days), median (IQR) | 1 (1-2) | 2 (1-7) |
| Hospital length of stay (days), median (IQR) | 3 (2-5) | 9 (6-14) |
| *Abbreviations: CSF, Cerebrospinal fluid; DCI, Delayed Cerebral Ischemia; GCS, Glasgow Coma Scale; ICH, Intracerebral hemorrhage; ICU, Intensive Care Unit; IQR, Interquartile Range; IVH, Intraventricular hemorrhage; PMH, Perimesencephalic Subarachnoid hemorrhage; WFNS, World Federation of Neurosurgical Societies*  **One patient* | | |
