# Supplementary material for: Spontaneous angiogram-negative subarachnoid hemorrhage: a retrospective single center cohort study
Source: Acta Neurochir (Wien). 2021 Dec 1;164(1):129–40. doi: 10.1007/s00701-021-05069-7 (PMC8761132; doi:10.1007/s00701-021-05069-7)
Supplement: Supplementary file 2 — (DOCX 711 kb) [file 701_2021_5069_MOESM2_ESM.docx]

| **Digital Supplemental 2 – Table 1**: Showing the five patients dying during follow-up | | | | | |
| --- | --- | --- | --- | --- | --- |
| **Patients dying** | **Age at admission** | **WFNS at admission** | **Modified Fisher grade admission** | **Days from admission to death** | **Cause of death** |
| Patient 1 | 59 | 3 | 4 | 6 | SAH |
| Patient 2 | 78 | 1 | 4 | 40 | SAH |
| Patient 3 | 43 | 4 | 4 | 111 | DKA and pneumonia |
| Patient 4 | 76 | 3 | 4 | 27 | SAH |
| Patient 5 | 66 | 5 | 2 | 11 | SAH |
| *Abbreviations: DKA, Diabetic ketoacidosis; SAH, Subarachnoid hemorrhage; WFNS, World Federation of Neurosurgical Societies* | | | | | |

**Re-bleed patient 1**

A 73-year-old woman was admitted due to acute headache, without neurological deficits. The head CT showed a typical perimesencephalic SAH (Figure 1). The CT-angiogram was negative. The patient was followed up in the hospital for four days in total and discharged without symptoms.

Two days after the discharge the headache intensified, and the patient's level of consciousness acutely dropped. She was readmitted to the hospital. The head CT showed a rebleeding extending into the brainstem (Figure 2). Repeat CT-angiograms, digital subtraction angiography (DSA), MRI and MR-angiography revealed no source of bleeding.


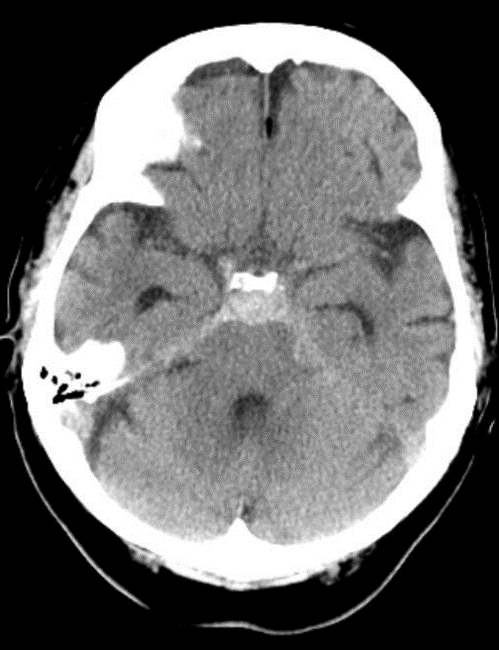


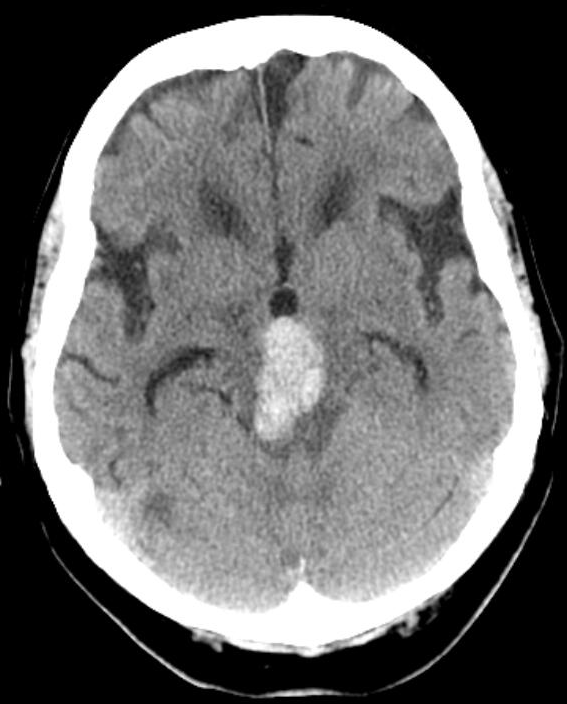


Figure 2: Head CT from the rebleed and readmission, six days after primary admission

Figure 1: Head CT from the primary bleed and admission

**Re-bleed patient 2**

A 39-year-old man was admitted due to sudden severe headache. The head CT showed blood in the right cerebellopontine corner (Figure 3). CT-angiogram and DSA showed no source of bleeding. The patient was discharged in good condition.

One year after the primary admission, the patient suffered once more from sudden headache and nausea. Patient presented to the emergency room and the head CT showed blood in the fourth ventricle and in the left cerebellopontine angle. (Figure 4). Repeat CT-angiograms, MR-angiograms and DSA did not reveal any source of bleeding.

Figure 4: Rebleed and readmission


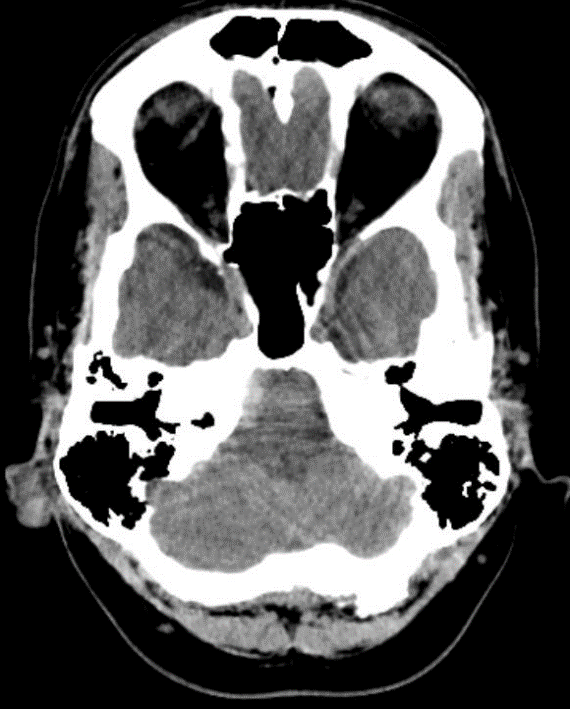

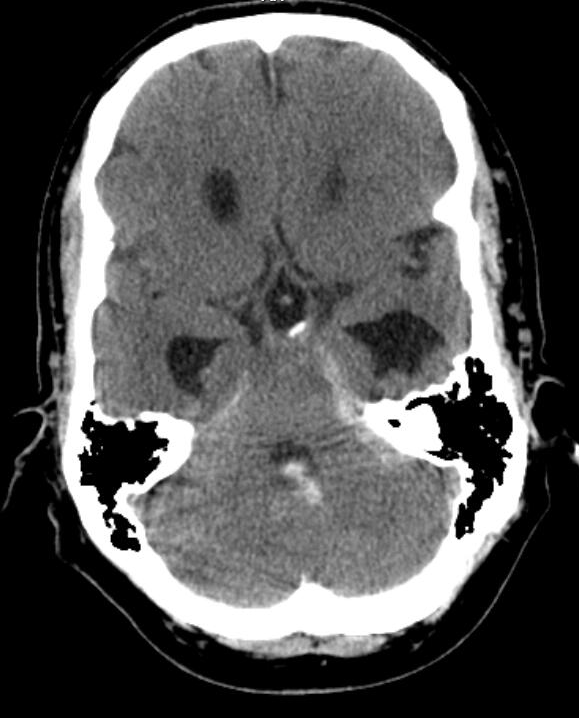


Figure 3: Primary admission and CT scan.
